# Indoor pollutant exposure is associated with heightened respiratory symptoms in atopic compared to non-atopic individuals with COPD

**DOI:** 10.1186/1471-2466-14-147

**Published:** 2014-09-10

**Authors:** Deepak A Kaji, Andrew J Belli, Meredith C McCormack, Elizabeth C Matsui, D’Ann L Williams, Laura Paulin, Nirupama Putcha, Roger D Peng, Gregory B Diette, Patrick N Breysse, Nadia N Hansel

**Affiliations:** Department of Medicine, School of Medicine, Johns Hopkins University, Baltimore, MD 21205 USA; Department of Environmental Health Sciences, Bloomberg School of Public Health, Johns Hopkins University, Baltimore, MD 21205 USA; Department of Pediatrics, School of Medicine, Johns Hopkins University, Baltimore, MD 21205 USA; Department of Biostatistics, Bloomberg School of Public Health, Johns Hopkins University, Baltimore, MD 21205 USA

**Keywords:** COPD, Atopy, Allergic sensitization, Pollutants, Particulate matter, PM, Indoor air, Susceptibility

## Abstract

**Background:**

Indoor particulate matter (PM) has been linked to respiratory symptoms in former smokers with COPD. While subjects with COPD and atopy have also been shown to have more frequent respiratory symptoms, whether they exhibit increased susceptibility to PM as compared to their non-atopic counterparts remains unclear. The aim of this study was to determine whether atopic individuals with COPD have greater susceptibility to PM compared to non-atopic individuals with COPD.

**Methods:**

Former smokers with moderate to severe COPD were enrolled (n = 77). PM_2.5_, PM with diameter <2.5 micrometers, was measured in the main living area over three one-week monitoring periods at baseline, 3, and 6 months. Quality of life, respiratory symptoms and medication use were assessed by questionnaires. Serum was analyzed for specific IgE for mouse, cockroach, cat, dog and dust mite allergens. Atopy was established if at least one test was positive. Interaction terms between PM and atopy were tested and generalized estimating equation analysis determined the effect of PM concentrations on health outcomes. Multivariate models were adjusted for age, sex, education, race, season, and baseline lung function and stratified by atopic status.

**Results:**

Among atopic individuals, each 10 μg/m^3^ increase in PM was associated with higher risk of nocturnal symptoms (OR, 1.95; P = 0.02), frequent wheezing (OR, 2.49; P = 0.02), increased rescue medication use (β = 0.14; P = 0.02), dyspnea (β = 0.23; P < 0.001), higher St. George’s Respiratory Quality of Life score (β = 2.55; P = 0.01), and higher breathlessness, cough, and sputum score (BCSS) (β = 0.44; P = 0.01). There was no association between PM and health outcomes among the non-atopic individuals. Interaction terms between PM_2.5_ and atopy were statistically significant for nocturnal symptoms, frequency of rescue medication use, and BCSS (all P < 0.1).

**Conclusions:**

Individuals with COPD and atopy appear to be at higher risk of adverse respiratory health effects of PM exposure compared to non-atopic individuals with COPD.

## Background

Chronic obstructive pulmonary disease (COPD) is characterized by progressive airflow limitation [1] and is a serious public health concern as it is the third leading cause of death in the United States [2]. COPD manifests after extended inhalation exposure to toxic agents such as cigarette smoke [3], and continued exposure among those with established disease is associated with worse disease severity [1]. We have previously shown that indoor air pollution, particularly particulate matter less than 2.5 micron in diameter (PM_2.5_), even at relatively low concentrations observed in US homes, was associated with increased respiratory symptoms and risk of severe COPD exacerbations in former smokers with moderate to severe COPD [4]. In addition, the presence of allergic sensitization has also been linked to worse respiratory symptoms in the same cohort of adults with COPD [5].

There is some evidence to suggest that allergic sensitization to aeroallergens is associated with increased susceptibility to the adverse health effects of PM in patients with asthma. For example, several studies suggest that atopic individuals have worse outcomes upon exposure to PM and other environmental pollutants compared to non-atopics with asthma [6–8]; however these results have not been consistent. For instance, some studies in asthma have not shown differential health effects of PM by atopic status; [9] other studies suggest that exposure to air pollutants is more deleterious in non-atopic than in atopic asthmatics [10, 11]. Whether atopic individuals with COPD are more susceptible to the effect of PM exposure compared to those without atopy has not previously been studied. The goal of this analysis was to investigate whether atopic individuals with moderate to severe COPD were more susceptible to the adverse effects of PM_2.5_ on respiratory health than non-atopic individuals.

## Methods

### Participant recruitment

Participants and methods were previously described [4]. Briefly, 84 former smokers with COPD meeting the following inclusion criteria were recruited from the Baltimore area: 1) age ≥ 40 years, 2) post bronchodilator FEV_1_ ≤ 80% predicted, 3) FEV_1_/FVC <70%, and 4) >10 pack years smoking, but having quit > 1 year prior to enrollment. Current non-smoking status was confirmed by requiring an exhaled carbon monoxide level ≤6 ppm at the time of recruitment [12]. Participants reporting a history of asthma (n = 7) were excluded from the current analyses as previously done in Jamieson et al. [5]. Participants provided written informed consent and the Johns Hopkins Medical Institutional Review Board approved the protocol.

### Air quality assessment

Air sampling was performed for one week at baseline, 3 and 6 months in the main living area, identified as a room, other than the bedroom, where the participant reported spending the most time and the bedroom. Indoor air sampling for PM_2.5_ and nitrogen dioxide (NO_2_) was conducted as described previously [9]. The limit of detection (LOD) for PM_2.5_ was 0.64 μg/m^3^.

### Clinical evaluation

Clinic visits occurred between day 4 and 7 of the air monitoring period at baseline, 3 and 6 months. Validated questionnaires assessed quality of life (St. George’s Respiratory Questionnaire (SGRQ)) [13], dyspnea (Medical Research Council (MRC) dyspnea scale) [14], and respiratory health. Presence of cough or phlegm was determined by a positive response to either of the following questions from the American Thoracic Society Division of Lung disease (ATS-DLD) Questionnaire [15]: “Do you usually have a cough?” or “Do you usually bring up phlegm from your chest” at each visit and was dichotomized to “yes” or “no.” Subjects reported whether they experienced wheeze in the last four weeks and responses were dichotomized to “frequent” if they reported symptoms almost every day, several days a week, or a few days a month and “infrequent” if they responded only with respiratory infections, or not at all. Nocturnal symptoms defined as coughing or breathing that disturbs sleep was dichotomized to “yes” or “no”. Frequency of rescue medication use (0, 1, 2, 3 or >4 times daily) and symptoms as assessed by Breathlessness, Cough and Sputum Score (BCSS) [16] were assessed by daily diary. Responses were averaged over each one-week monitoring period. Spirometry, before and after albuterol, was performed according to American Thoracic Society (ATS) criteria [17, 18]. Serum was analyzed for specific IgE by ImmunoCAP (Phadia, ThermoFisher, USA) for mouse, cockroach, cat, dog and dust mite allergens. A participant was considered atopic if at least one test was at or above the level of detection (0.1 kUA/L).

Exacerbations over the duration of study were assessed by questionnaires at each clinic visit and by monthly telephone calls. Any exacerbation was defined as worsening respiratory symptoms requiring antibiotics, oral steroids or an acute care visit. Severe exacerbations were defined as worsening respiratory symptoms leading to an Emergency department (ED) visit or hospitalization.

### Statistical analysis

Descriptive statistics were analyzed using likelihood-ratio tests and t-tests, as appropriate. At each time point, the PM_2.5_ concentrations were used as exposure variables in generalized estimating equations models [15], in order to account for the correlation arising from repeated measures of the outcomes over time; adjusting for age, sex, education, season (spring/summer vs. fall/winter) and pre-bronchodilator % predicted FEV_1_. In sensitivity analysis, we also included the use of inhaled corticosteroids (ICS) as a confounding variable. Interaction terms were used in the final models to formally test for interactions between PM_2.5_ and atopy. Because significant interactions between PM_2.5_ and atopy were identified, analyses investigating the association between PM_2.5_ and COPD outcomes were stratified by atopic status. To evaluate the effect of PM_2.5_ on respiratory health, continuous and binary outcomes were analyzed using linear and logistic regression models, respectively, with PM_2.5_ included as a continuous predictor. All models with a generalized estimating equations approach assumed exchangeable correlations. The primary analyses included effects of the main living area as this was found to be the most important area of exposure in regards to COPD outcomes [4]. Investigation of interactions with atopy and associations between NO_2_ and bedroom PM_2.5_ with health outcomes were included as secondary analyses.

All analyses were performed with StataSE statistical software, version 11.0 (Stata Corp, College Station, TX). A *P* value less than 0.05 was considered statistically significant for main effects and a *P* value less than 0.10 was considered statistically significant for interactions for modestly sized populations, as previously done and recommended by Selvin et al. [19].

## Results

### Baseline participant characteristics and pollutant concentrations

All participants (n = 77) had moderate or severe COPD with a mean baseline post-bronchodilator FEV_1_ % predicted of 52.3%. As previously published, a third (30%) of individuals were atopic (17 tested positive to cockroach, 16 to house dust mite, 7 to dog, 6 to cat and 1 to mouse allergen). At baseline, atopic participants with COPD were significantly more likely to report the presence of wheeze, nocturnal cough, and health care utilization in the previous one year compared to non-atopic subjects [5]. There was no difference in reported baseline quality of life (SGRQ score), dyspnea (MMRC score), or prevalence of common comorbidities (Table 1).

**Table 1:** **Baseline participant characteristics**

Participant characteristics	Non atopic (n = 54)	Atopic (n = 23)	p
**Age, mean (SD)**	69.3 (7.1)	69.7 (7.1)	0.86
**Gender, % male**	63	57	0.60
**Race, (%)**			
Caucasian	89	83	0.71
Black/African American	9	13	
Other	2	4	
**Education, (%)**			
< High School	19	22	0.63
High School	24	9	
Some College	30	39	
Bachelor’s Degree	13	13	
At least some Graduate School	15	17	
**Smoking History, mean (SD)**			
Pack Years	56.8 (28.4)	60.7 (31.0)	0.60
Last Cigarette (Years Since)	13.0 (9.7)	13.4 (8.0)	0.85
**Baseline Health Status**			
Post Bronchodilator FEV_1_% predicted, mean (SD)	52.4 (16.11)	51.9 (17.29)	0.89
Pre FEV_1_/FVC	0.50 (0.10)	0.52 (0.10)	0.45
Post FEV_1_/FVC	0.51 (0.10)	0.54 (0.11)	0.28
Bronchodilator reversibility, (%)	31.5	30.4	0.93
Chronic Bronchitis (%)	39	35	0.73
Emphysema (%)	57	70	0.32
SGRQ, mean (SD)	38.6 (17.6)	42.0 (20.7)	0.47
MMRC, mean (SD)	2.6 (1.0)	2.5 (1.2)	0.63
**Medication list, (%)**			
Long acting beta agonist (LABA)	8	4	0.61
Inhaled Corticosteroids (ICS)	19	5	0.12
ICS/LABA combination	44	65	0.10
Long acting muscarinic antagonist	37	43	0.60
Nasal steroids	6	9	0.61
Leukotriene modifiers	8	9	0.88
Theophylline	2	9	0.16
Antihistamine	2	9	0.16
**Comorbidities, (%)**			
Congestive heart failure	6	9	0.61
Diabetes	19	26	0.45
Cancer	19	22	0.74
Myocardial infarction	11	22	0.22
Hypertension	59	61	0.90
Kidney disease	2	0	0.51

Please note some of the data from the above table were previously published in a manuscript from the CODE cohort [5].

BCSS = Breathlessness, Cough, and Sputum Scale.

SGRQ = St. George’s Respiratory Questionnaire.

MMRC = Modified Medical Research Council.

FEV_1_ = Forced expiratory volume in 1 second.

FVC = Forced vital capacity.

SD = Standard deviation.

Atopic and non-atopic participants reported spending similar amounts of time indoors (90% and 92%, respectively). At baseline, the median PM_2.5_ (IQR) concentrations tended to be higher in homes of atopic individuals (12.3 (4.7, 26.8) μg/m^3^ vs. 9.0 (2.6, 29.3) μg/m^3^, p = 0.07). There were no significant differences in regards to type of housing, heating, or cooking or presence of air nicotine or report of other smokers in the home between those with and without atopy (data not shown).

### Association of indoor pollutant concentrations and respiratory health

In bivariate analyses, increasing PM_2.5_ concentrations in the main living area were associated with increased frequency of wheeze, rescue medication use, nocturnal symptoms and worse quality of life (SGRQ) among atopic individuals (Table 2). Among the non-atopic individuals, there were no statistically significant associations between PM and respiratory outcomes.

**Table 2:** Bivariate association of indoor pollutant concentrations* and respiratory health

Bivariate analysis	Non-atopic participants (n = 54)			Atopic participants (n = 23)		
	β	p	95% CI	β	p	95% CI
MMRC (dyspnea)	0.11	0.31	[−0.11,0.33]	**0.21**	**0.01**	**[0.06,0.37]**
Rescue medication use	0.06	0.49	[−0.11,0.24]	**0.18**	**0.03**	**[0.02,0.34]**
BCSS	−0.28	0.13	[−0.65,0.09]	0.32	0.09	[−0.05,0.69]
SGRQ	1.04	0.47	[−1.77,3.85]	**2.15**	**0.05**	**[0.03,4.27]**
	**OR**	**p**	**95% CI**	**OR**	**p**	**95% CI**
Wheeze	1.38	0.14	[0.90,2.11]	**2.25**	**0.01**	**[1.22,4.14]**
Nocturnal symptoms	1.00	0.98	[0.55,1.80]	**1.76**	**0.04**	**[1.02,3.01]**
Cough	0.76	0.27	[0.46,1.24]	1.11	0.50	[0.82,1.51]
Phlegm	1.09	0.67	[0.72,1.66]	1.15	0.37	[0.84,1.57]
Severe exacerbations	0.89	0.81	(0.35, 2.28)	1.27	0.21	(0.87, 1.83)

BCSS = Breathlessness, Cough, and Sputum Scale.

SGRQ = St. George’s Respiratory Questionnaire.

MMRC = Modified Medical Research Council.

*ORs and coefficients represent difference in stated outcomes for every 10 point change in PM_2.5_.

Bold values represent statistically significant associations (p <0.05).

**Figure 1:**
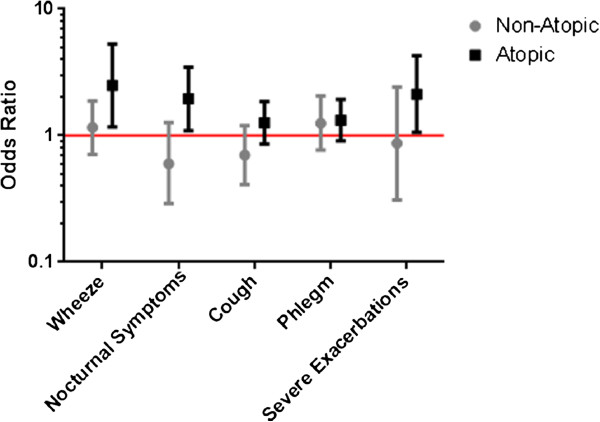
**Differential health effects of PM on respiratory health among atopic and non-atopic former smokers with COPD.**

Similarly, after adjustment for confounders in multivariate analyses, indoor PM_2.5_ in the main living area was significantly associated with respiratory health outcomes among atopic individuals but not among non-atopic individuals (Table 3) (Figure 1). Specifically, among atopic individuals, increasing PM_2.5_ concentrations were significantly associated with increased risk of nocturnal symptoms (OR 1.95, p = 0.02), BCSS scores (β = 0.44, p = 0.01) and frequency of rescue medication use and (β = 0.14, p = 0.02), with atopy significantly modifying the effect of PM exposure (all p interaction values < 0.1). In addition, a 10 μg/m^3^ increase in PM_2.5_ concentrations was also associated with increased dyspnea (higher MMRC score, β = 0.23, p < 0.001), worse quality of life (higher SGRQ score, β = 2.55, p = 0.01) and higher likelihood of having frequent wheeze (OR = 2.49, p = 0.02) among atopic individuals. PM_2.5_ was not significantly associated with health outcomes in non-atopic individuals (Table 3). Adjusting for the use of ICS did not substantially change the results (data not shown).

**Table 3:** Multivariate analyses of association of indoor pollutant concentrations and respiratory health*

Multivariate analysis	Non-atopic participants (n = 54)			Atopic participants (n = 23)			int(p)
	β	p	95% CI	β	p	95% CI	
MMRC	0.08	0.52	[−0.16, 0.32]	**0.23**	**<0.001**	**[0.09, 0.34]**	0.62
Rescue medication use	0.01	0.90	[−0.18, 0.20]	**0.14**	**0.02**	**[0.02, 0.26]**	<0.001
BCSS	−0.30	0.13	[−0.70, 0.09]	**0.44**	**0.01**	**[0.11, 0.76]**	0.08
SGRQ	1.06	0.45	[−1.67, 3.80]	**2.55**	**0.01**	**[0.69, 4.41]**	0.44
	**OR**	**p**	**95% CI**	**OR**	**p**	**95% CI**	
Frequent wheeze	1.16	0.56	[0.71, 1.88]	**2.49**	**0.02**	**[1.17, 5.30]**	0.17
Nocturnal symptoms	0.60	0.19	[0.29, 1.27]	**1.95**	**0.02**	**[1.09, 3.48]**	0.03
Cough	0.70	0.20	[0.41, 1.20]	1.26	0.23	[0.86, 1.85]	0.28
Phlegm	1.25	0.37	[0.77, 2.05]	1.32	0.15	[0.91, 1.93]	0.79
Severe exacerbations	0.87	0.79	[0.31, 2.42]	**2.12**	**0.03**	**[1.06, 4.27]**	0.63

BCSS = Breathlessness, Cough, and Sputum Scale.

SGRQ = St. George’s Respiratory Questionnaire.

MMRC = Modified Medical Research Council.

*ORs and coefficients represent difference in stated outcomes for every 10 point change in PM_2.5_.

Bold values represent statistically significant assiciations (p <0.05).

Though, bedroom PM_2.5_ concentrations were not associated with COPD outcomes in main effect analyses previously published [4], we found statistically significant interactions between bedroom PM_2.5_ and atopy for both nocturnal respiratory symptoms and risk for severe exacerbations. Specifically, the relationship between bedroom PM_2.5_ concentrations and nocturnal respiratory symptoms differed by atopic status (OR 1.05, p = 0.32 for atopic vs. OR 0.94, p = 0.21 non-atopic) as did the risk for severe exacerbations (OR 1.2, p = 0.04 for atopic vs. OR 0.81, p = 0.15 for non-atopic, interaction p-value for both comparisons <0.1). There were no statistically significant interactions between bedroom PM_2.5_ and atopy for other outcomes.

A 20 point increase in NO_2_ concentrations in the main living was more likely to be associated with increased risk of cough in atopic individuals (OR 3.21, p = 0.08) compared to non-atopic individuals (OR 0.67, p = 0.35), p-interaction 0.09. There were no significant interactions between bedroom or main living area NO_2_ concentrations and atopy for the remaining health outcomes (data not shown).

## Discussion

To our knowledge, this is the first study investigating whether the presence of atopy modifies the effect of PM exposure on respiratory health in individuals with COPD. It has been previously shown that atopy is prevalent in populations of COPD and is linked to worse respiratory symptoms [20, 21]; and that higher PM exposure is associated with worse respiratory outcomes in former smokers with COPD [4]. Our results show that atopic individuals with COPD may be more susceptible to the effects of PM exposure than non-atopic individuals. In particular, higher indoor PM concentration was linked to increased daytime and nocturnal respiratory symptoms and more frequent rescue medication use among atopic individuals but not among non-atopic individuals. This data suggest that atopic patients living with COPD may exhibit a differential response to particulate matter compared to their non-atopic counterparts.

Studies evaluating whether allergic sensitization to aeroallergens is associated with increased susceptibility to the adverse health effects of pollutant exposure in patients with other chronic lung diseases, such as asthma have been conducted however results have been inconsistent. Some epidemiologic studies, similar to our current study in COPD, have found that atopic individuals with asthma may be more susceptible to the effects of PM. For example, in a small study with 19 asthmatic children, Delfino et al. showed that PM_2.5_ exposure was associated with a larger reduction in lung function in children allergic to indoor allergens as compared to those that were non-allergic [22]. It has also been suggested that tobacco smoke exposure is linked to worse respiratory symptoms in atopic children compared to non-atopic children with asthma [23]. In regards to NO_2_, epidemiological and experimental studies also suggest that atopic status may modify susceptibility to NO_2_ exposure in individuals with asthma, but the results have also been inconsistent [24]. Some studies suggest that there is no differential effect of air pollution exposure on atopic or non-atopic individuals [9] and others have shown that non-atopic individuals may be more susceptible [7, 25–27].

There are several potential mechanisms by which the presence of atopy may increase susceptibility to PM exposure. For example, several studies have shown that PM acts as a carrier to some aeroallergens (e.g., cat, dog, birch pollen, and house dust mite allergen) [28, 29]. Therefore, the link between higher PM concentrations and health effects may not be due to the direct effects of particulate matter but to the associated increased allergen exposure in sensitized individuals. Furthermore, PM may work as an adjuvant with allergens to incite inflammatory pathways [30]. There are several human and animal experimental studies which support a possible synergistic effect between air pollution and allergen exposure. Individuals with asthma have been shown to experience a greater drop in lung function upon exposure to allergen and air pollutants compared to those exposed to allergen without air pollutants [6, 31]. Alberg et al. showed that a combined dosage of PM and Ova allergen injected into mice lead to higher serum anti-Ova IgE concentrations compared with an injection of the allergen Ova alone [32].

In addition to PM being a surrogate for allergen exposure or being linked to a heightened response to allergen exposure, those with atopy may have an inherent predisposition to a heightened response to PM itself. Atopy has been linked to increased underlying eosinophilic airway and systemic inflammation as well as increased bronchial hyper-responsiveness (BHR) even in individuals without asthma [33–35]. This underlying eosinophilic inflammation or increased BHR may predispose individuals to the adverse health effects of pollutant exposure. For example, ozone exposure worsened BHR in a dose dependent fashion in atopic guinea pigs, but did not induce airway hyper-responsiveness in the non-atopic guinea pigs [36]. Being an observational study, we were unable to identify mechanistic underpinnings for our associations which merits further examination.

The moderate size of the study population limits our ability to detect statistically significant interactions and raises the possibility of type II error, which underscores the need to test our hypotheses in future studies. Despite this, we found statistically significant interactions for several outcomes and a consistent trend of stronger adverse effect of PM concentrations across all outcomes among atopic individuals lending validity to our results that atopic individuals with COPD are more susceptible the effects of PM than non-atopic individuals. Allergen concentrations on PM filters were not available and therefore we were unable to determine whether higher allergen concentrations in PM were linked to worse health outcomes. Furthermore, our assessment of atopic status was limited to perennial allergens and we were not able to investigate whether increasing number of allergic sensitizations or specific sensitizations portends greater risk to the adverse health effects of PM on patients with COPD.

## Conclusion

Our study demonstrates that increasing particulate matter concentrations are more strongly associated with worsening respiratory symptoms for atopic COPD patients than for non-atopic COPD patients. As this may be the first study to look at the importance of allergic sensitization as a modifier of particulate matter as it pertains to COPD, very little clinical guidance currently exists for the differential management of atopics versus non-atopics with COPD. Additional research is needed to support the findings of this study in a larger sample size in order to further characterize the role of an allergic phenotype in COPD and to determine strategies to reduce PM and improve outcomes in this important population.

## Authors’ information

Deepak A Kaji and Andrew J Belli represents two first authors.

## Abbreviations

COPD: Chronic obstructive pulmonary disease
PM: Particulate matter
BCSS: Breathlessness, cough, and sputum scale
SGRQ: St. George’s respiratory questionnaire
MRC: Medical research council
FEV_1_: Forced expiratory volume in 1 second
FVC: Forced vital capacity
IQR: Interquartile range
SD: Standard deviation
OR: Odds ratio
NO_2_: Nitrogen dioxide.
